# Mechanical Thrombectomy via Transbrachial Approach in the Emergency Management of Acute Ischemic Stroke Patients with Aortic Pathologies: Our Experience and Literature Review

**DOI:** 10.3390/jpm14020216

**Published:** 2024-02-18

**Authors:** Aida Iancu, Raluca Tudor, Dana Simona Chita, Catalin Juratu, Anca Tudor, Florina Buleu, Daian Popa, Silviu Brad

**Affiliations:** 1 Department of Radiology, “Victor Babes” University of Medicine and Pharmacy, E. Murgu Square no. 2, 300041 Timisoara, Romania; aida.parvu@umft.ro (A.I.); catalin.juratu@umft.ro (C.J.); bradsilviu@yahoo.com (S.B.); 2 County Emergency Clinical Hospital “Pius Brinzeu”, 300732 Timisoara, Romania; buleu.florina@gmail.com; 3 Department of Neurology, “Victor Babes” University of Medicine and Pharmacy, E. Murgu Square no. 2, 300041 Timisoara, Romania; 4 Department of Neurology, Faculty of General Medicine, “Vasile Goldis” Western University of Arad, 310025 Arad, Romania; chita.dana-simona@uvvg.ro; 5 Department of Functional Sciences, “Victor Babes” University of Medicine and Pharmacy, E. Murgu Square no. 2, 300041 Timisoara, Romania; atudor@umft.ro; 6 Department of Cardiology, “Victor Babes” University of Medicine and Pharmacy, E. Murgu Square no. 2, 300041 Timisoara, Romania; daian-ionel.popa@umft.ro; 7 Department of Surgery, Emergency Discipline, “Victor Babes” University of Medicine and Pharmacy, 300041 Timisoara, Romania

**Keywords:** acute ischemic stroke emergency management, aortic pathologies, mechanical thrombectomy, case report, literature review

## Abstract

Study design: Mechanical thrombectomy (MT) via the transbrachial approach (TBA) is a very rare option used in cases of patients with aortic pathologies and acute ischemic stroke (AIS) due to the insufficient evidence in the literature, the difficulty from a technical point of view and the result of this technique influenced by the complications that frequently accompany it. Background: Only a few cases of patients with aortic pathologies and acute ischemic stroke where MT via TBA were reported in the literature, and its application in the emergency management of AIS has still not been dealt with in detail. Objectives: Out of a need to clarify and clearly emphasize the effectiveness of this approach in emergency MT via TBA in patients with AIS and aortic pathologies, this literature review and case report has the following objectives: the first one is the presentation of an emergency MT via transbrachial approach performed in a 44-year-old patient with AIS and diagnosed aortic coarctation during transfemural approach (TFA), with successful reperfusion in our department and the second one is to review the cases reports of patients with different aortic pathologies and AIS reperfusion therapy performed by MT via TBA from the literature. Methods: A total of nine cases (one personal case and eight published cases) were revised in terms of aortic pathologies type, reperfusion therapy type, and the complication of both mechanical thrombectomy and local transbrachial approach. Results: Mechanical thrombectomy through the transbrachial approach was the first choice in more than half of these cases (55.55%, n = 5 cases) in the treatment of acute ischemic stroke in the presence of previously diagnosed aortic pathologies. In one-third of all cases (33.33%, n = 3, our case and 2 case reports from the literature), the transbrachial approach was chosen after attempting to advance the guiding catheter through the transfemoral approach and intraprocedural diagnosis of aortic pathology. In only one case, after an ultrasound evaluation of the radial artery that showed a monophasic flow, MT was performed via TBA. Local transbrachial complication was reported in one case, and in two other cases, it was not stated if there were such complications. Hemorrhagic transformation of AIS was reported in two cases that underwent MT-only cerebral reperfusion via TBA, one with acute aortic dissection type A and our case of previously undiagnosed aortic coarctation. In the cases in whom short and long-term follow-up was reported, the outcome of treatment, which was not exclusively endovascular (77.77% cases with only MT and 33.33% with association of first thrombolysis and after MT), was good (six from nine patients). In two case reports, the outcomes were not stated, and one patient died after a long hospitalization in the intensive care unit from respiratory complications (our patient). Conclusions: Being a clinical emergency, acute ischemic stroke requires urgent medical intervention. In patients with aortic pathologies, where acute ischemic stroke emergency care is a challenge, mechanical thrombectomy via the transbrachial approach is a safe alternative method for cerebral reperfusion.

## 1. Introduction

Mechanical thrombectomy (MT) has proven its importance in improving outcomes after acute ischemic stroke (AIS) over the past decade, a fact increasingly emphasized by large, multi-center, randomized, controlled clinical trials [1,2,3,4]. According to these, the conventional transfemoral approach is the basic approach for MT due to optimal intraoperative catheterization and immediate access to large-diameter devices (which provides more confidence when inserting high-profile catheters/sheaths) or due to the higher frequency complications of other vascular approaches or because of the absence of formal training in medical schools [1,2,3,4,5]. 

However, TFA has some reported limitations. In patients with type III aortic arch, extensive atherosclerotic disease involving the arch and descending aorta, ilio-femoral athero-occlusive disease, atypical branching patterns, coarctation or dissections of the aorta that limit blood flow, cannulation of the aortic arch vessels using TFA may be a challenge [6]. Apart from these limitations, access-site complications such as groin hemorrhages and hematomas, pseudoaneurysms, retroperitoneal hematomas, peripheral artery occlusions, femoral nerve injuries, and access-site infections. All these complications have been reported (5.13% in non-randomized control trials, while in randomized control trials the rate is 2.78%) in a systematic review that included 16 randomized clinical trials (RCTs) and 17 non-randomized cohort studies [7].

When faced with challenging situations in acute ischemic stroke emergency management where MT via TFA may pose difficulties, it becomes essential to utilize alternative puncture techniques like transradial, transbrachial, or direct carotid puncture to successfully achieve vascular and thrombic access, despite the limited documentation surrounding their efficacy [8].

In other to provide clarity and emphasize the effectiveness of utilizing the transbrachial approach in emergency mechanical thrombectomy for patients with acute ischemic stroke and aortic pathologies, this literature review and case report aims to achieve the following objectives: firstly, to present a case from our own experience involving a 44-year-old hypertensive patient with AIS and previously undiagnosed aortic coarctation, in which we successfully performed emergency MT via the transbrachial approach; and secondly, to review case reports from the literature on patients with various aortic pathologies and acute ischemic stroke who underwent mechanical thrombectomy via TBA.

## 2. Case Report and Literature Review

### 2.1. Case Presentation

A 44-year-old man with a history of essential hypertension was brought into the emergency department (ED) by an ambulance with a doctor with left hemiparesis, inability to talk, and anarthria with an onset of approximately 150 min. The patient’s stroke-related neurological deficit assessment with the National Institute of Health Stroke Scale (NIHSS) was 15. 

**Figure 1:**
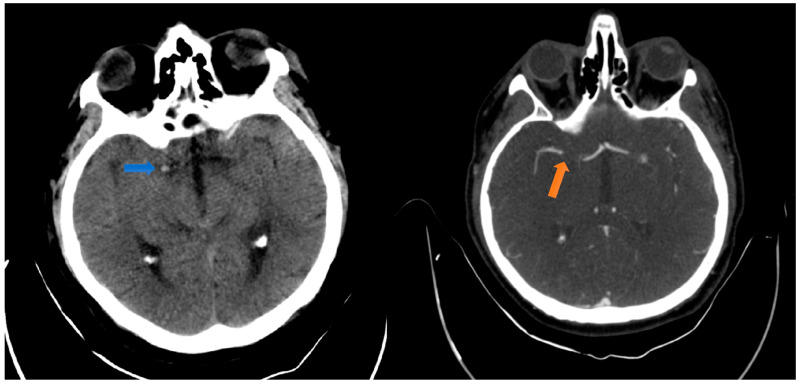
The left image displays the initial CT scan of the head, revealing no significant indications of ischemic or hemorrhagic events. The PC-ASPECTS score was 10. A hyperdense right middle cerebral artery is visible, indicated by a blue arrow. The right image shows a multiphase head angiography-CT, confirming the presence of a thrombus at the level of the MCA as segment M1, as indicated by a red arrow.

The non-contrast computed tomography (CT) scan and the CT angiography (CTA) of the head and neck (Figure 1) multiphase performed at the ED admission detected a dense right middle cerebral artery (MCA) sign with a right M1 occlusion visibility from the emergence of the carotid artery over a length of approximately 1 cm and reinjection into M2. The dense MCA sign has a crucial role in diagnosing acute stroke. It often becomes visible on CT imaging before other signs of infarct, indicating an occlusion of a large artery within the brain and the resulting infarct [9].

No clinically significant stenotic lesions were identified in the common carotid artery or internal or external carotid arteries bilaterally. The right vertebral artery was hypoplastic, with the right vertebral artery dominant and the bilateral basilar artery permeable. No acute cerebral hemorrhagic lesions were constituted, with an Alberta Stroke Program Early CT Score (PC-ASPECTS) of 10. All laboratory analyses were normal.

Under general anesthesia, emergency MT was conducted at almost 6 h following the onset of stroke symptoms. A transfemoral approach was obtained in order to perform mechanical thrombectomy. During the advancement with the guide catheter, aortic coarctation was discovered, which remained undiagnosed until that moment, and the initial standard femoral artery approach was abandoned. 

**Figure 2:**
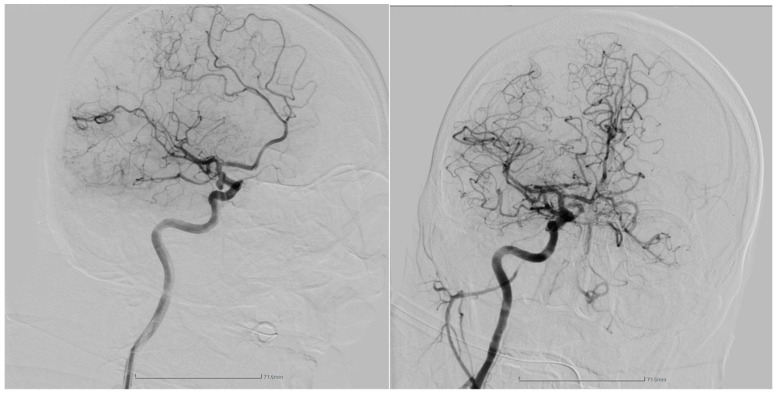
The lateral view of the digital subtraction angiography shows the occlusion of the right anterior cerebral artery (MCA) before the endovascular thrombectomy (left image). After the procedure (right image), a complete revascularization of the previously occluded arterial territory was successfully achieved.

The procedure involved secondly a transbrachial approach using an 8F sheath. Selective cannulation of the right common carotid artery was performed, followed by inserting a 0.035-inch guidewire. With a single retrieval attempt using a 4 × 20 mm stent, the thrombus was successfully extracted. Puncture resulted in complete revascularization of the occluded arterial territory (see Figure 2). Throughout the procedure, no clinical or technical complications were encountered immediately.

**Figure 3:**
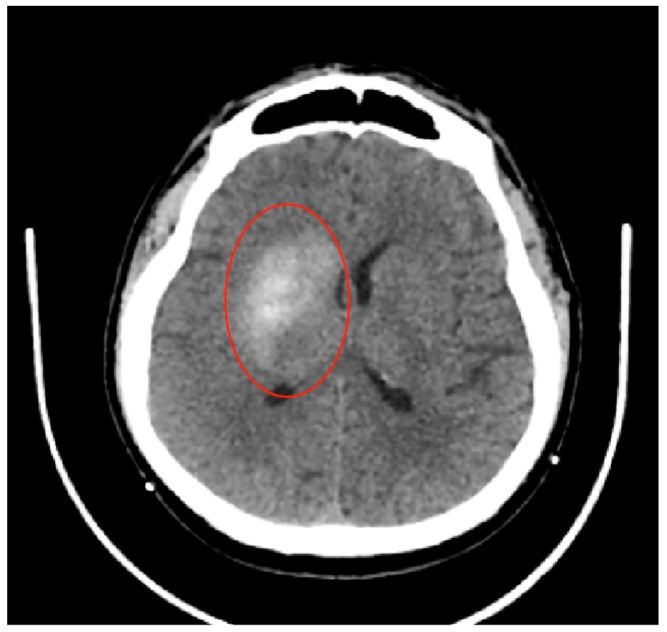
Head CT scan performed at 24 h after mechanical thrombectomy showed residual contrast substance in the nucleus basalis after revascularization—without established cerebral ischemia (red circle). The PC-ASPECTS was 10.

With unassisted breathing, full awareness, and orientation, the patient was relocated to the neurology department. A day following the mechanical thrombectomy procedure, the patient experienced a significant improvement in neurological function. A subsequent CT scan revealed no cerebral ischemia in the revascularized area, scoring a perfect ASPECT 10 (Figure 3). 

**Figure 4:**
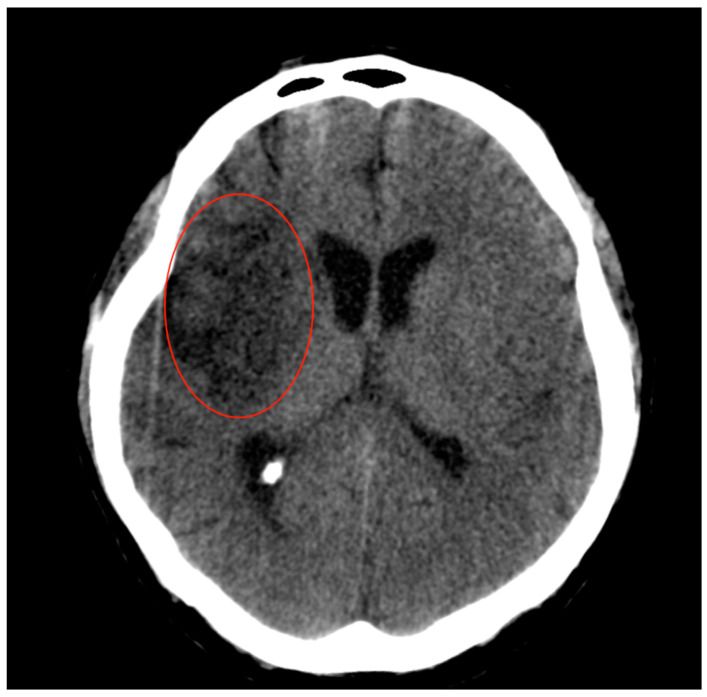
Head CT scan perform at 7 days after MT showed ischemia in the vascularized territory (lentiform nucleus and in the fronto-temporal-insular right lobe) (red circle).

At the head CT examination 7 days post-procedural (Figure 4), compared to the previous examinations, it was observed the disappearance of the contrast substance residues from the nucleus basalis, as well as an extended ischemic hypodensity in the superficial right MCA (fronto-temporal) and in the lateral lenticulostriate arteries; ischemic hypodensity also occurred at the level of the right caudate nucleus. 

On the morning of the 8th admission day, he was found to have a sudden onset of conscious disturbance and left hemiparesis with an NIHSS score of 22. EKG showed atrial fibrillation with rapid response. Embolic occlusion due to atrial fibrillation was highly suspected, and the patient received anticoagulant therapy with good neurological evolution. The patient developed respiratory complications due to a severe pulmonary infection. On the 31st day of hospitalization, the patient had a cardiac arrest that was successfully resuscitated, followed by patient transfer to the intensive care unit. During hospitalization in the intensive care unit, the patient had a fluctuating evolution, and despite the treatment (medication, kinetotherapy, hydroelectrolyte rebalance solutions), the patient’s condition was deteriorating and required vasopressor support and mechanical ventilation. Six days after admission into the intensive care unit, the patient had cardiac arrest through asystole and did not respond to resuscitation maneuvers.

### 2.2. Literature Review

In general, the aortic arch has three major branches from proximal to distal: the brachiocephalic trunk, the left common carotid artery, and the left subclavian artery. These branches supply blood to the head, neck, and upper chest. The most common branching pattern, known as the standard arch, consists of the right brachiocephalic, left common carotid, and left subclavian arteries from right to left. According to a systematic review and meta-analysis of 51 articles (n = 23,882), the prevalence of standard arches is 80.9% [10]. Inanc et al. [11] identified three variations in the standard arch. The most common type, Type I, was found in 60.7% of patients, Type II (the bovine aortic arch) was seen in 34.3% of patients, and Type III was seen only in 4.8% of patients. Among the most common congenital malformations that occur in approximately 1% of all live births, aortic coarctation represents a proportion of 6–8% of these, according to the study performed by Hoffman and Kaplan [12].

Individuals with variations in the aortic arch and aortic coarctation may be more susceptible to atherosclerosis, leading to an increased risk of stroke due to the potential formation of blood clots. The alteration of hemodynamics could potentially occur due to the abnormal origin of arteries from the aortic arch or narrowing of the aorta, as hypothesized by Satti et al. [13].

Aortic pathologies present challenges during endovascular acute stroke treatment procedures, as highlighted in a study by Ribo et al. in 2013. The presence of these pathologies leads to prolonged procedure time and makes tasks such as carotid artery cannulation and stenting particularly difficult [14].

In fact, in patients with aortic pathologies and AIS, it is unclear how often alternative access is used in clinical practice and whether outcomes differ between patients who underwent MT through alternative (radial, carotidian, or brachial) versus transfemoral access [15].

Further, we conducted a literature review. Inclusion criteria were as follows: papers reporting cases with mechanical thrombectomy via transbrachial approach in patients with acute ischemic stroke and aortic pathologies, written in the English language and with full-text availability. We performed a comprehensive literature search of four electronic databases (PubMed, Scopus, Web of Science, Google Scholar) from inception until the first of November, 2023, using this search query (“Transbrachial” OR “TBA”) AND (“case report” or ”case reports”) AND (“Mechanical thrombectomy” OR “MT” OR “Endovascular thrombectomy” OR “acute stroke”) AND (“aortic pathologies” OR “aortic”). All duplicates were removed, and all references in the included case reports were screened manually for any eligible studies. Additionally, we cross-referenced the bibliographies of retrieved articles and review papers to ensure that we captured all case reports that met our research terms. Moreover, we performed a manual search for conference abstracts and/or posters or case reports in other databases to include in our review.

Ultimately, eight articles met the criteria for inclusion in this review, and one was excluded due to the unavailability of our data regarding the criteria followed (lack of concrete data about the included patients). Notably, all seven articles were case reports discussing TBA in patients with AIS and aortic pathologies, as summarized in Table 1.

## 3. Discussion

To the best of our knowledge, a literature review of aortic pathologies type, reperfusion therapy type, and the complication of both mechanical thrombectomy and local transbrachial approach in patients with acute ischemic stroke has not been previously published in the literature. Only a few cases of patients with aortic pathologies and acute ischemic stroke where MT via TBA was performed are reported [16,17,18,19,20,21,22], and this technique application in emergency management of AIS reperfusion has still not been dealt with in detail. So, we conducted a literature review, and after we analyzed these similar cases and from our case experience, we believe that TBA can be a feasible alternative route in AIS patients with aortic pathologies where the transfemoral approach cannot be used.

In interventional neuroradiology, the transfemoral artery approach remained the preferred arterial access site for most of the doctors who performed MT [6]. Only in cases where TFA has limitations [6], like coarctation of the aorta, a congenital malformation that typically presents in childhood, the other approach sites are used. Clinically, these patients present hypertension that appears in youth with a difference in blood pressure between the upper and lower extremities [23].

We present our case, where, unusually, the patient’s aortic pathologies remained undiagnosed until his fortieth decade. Moreover, although he was hypertensive, with values most often difficult to control, only at the time of performing the MT via transfemoral approach the aortic coarctation was diagnosed. During the procedure, the interventional neuroradiologist decided to use the transbrachial approach for MT in order not to delay reperfusion management and for the patient not to leave the MT time window (<6 h). In this particular case, anticipating the possibility of inserting a larger caliber catheter (8F) for aspiration, the transbrachial approach was chosen instead of the radial. Also, based on his experience, the interventional neuroradiologist decided on this approach. 

It is uncommon to incidentally discover aortic coarctation in adult patients, especially in association with AIS, particularly in adult and elderly patients who have not undergone correction for coarctation. The survival rate for patients with aortic coarctation is generally low [24], and there is ongoing debate regarding the best management strategies for such cases, particularly when other conditions like AIS complicate it. 

When combined with secondary arterial hypertension, aortic coarctation increase the likelihood of developing premature coronary artery disease, aortic aneurysms, and cerebrovascular diseases, which not only affect prognosis but also have a significant impact on morbidity and disability [25]. The current guidelines strongly recommend the early detection and proactive management of aortic coarctation [23]. Despite advancements in surgical and interventional treatments for aortic coarctation, there have been reported cases of neurological complications, particularly paraplegia. These complications can arise from direct injury to spinal segmental arteries or inadequate oxygen supply to the spinal cord during the surgery correction process, leading to palsy or paralytic syndromes [26]. 

Two years ago, Trenk et al. [25] analyzed the results of a nationwide report based on 11,907 patients with coarctation of the aorta hospitalized between 1997 and 2015 in England. Late neurological complications (e.g., cerebral infarction and subarachnoid hemorrhage) occurred in 225 patients out of almost 150,000 patients followed annually during the study period (rate of 0.15%/patient-year). Subarachnoid hemorrhage occurred in 59.4% (n = 37 patients, 22 men) and at a median age of 28.7 years (IQR 20.2–44.5 years). Of these, prior to inclusion, only one patient had a diagnosis of cerebral arterial aneurysm. Ischemic stroke occurred in 188 patients. The median age at which they developed acute AIS was 56.1 years (IQR 43.6–68.1 years). Thirteen patients (6.9%) died during hospital admission, and the one-year mortality after stroke was 20%. Univariate conditional logistic regression analysis compared with patients matched for age, sex, and ethnicity showed that hypertension (odds ratio 4.00, *p* = 0.0014), dyslipidemia (odds ratio 4.50, *p* = 0.007), active smoking (odds ratio 11.00, *p* = 0.02), and diabetes mellitus (odds ratio 9.00, *p* = 0.037) were significantly related to the risk of AIS development. In multivariate analysis, only hypertension and smoking remained significantly related to the risk of ischemic stroke.

In the literature, there are few studies or case series studies that analyzed the transbrachial approach in emergency management of AIS [3,27,28]. When the brachial approach was compared with transradial or femoral approaches, it was noted to be more likely to have minor complications, with an overall access-site complication rate for noncoronary procedures of 10%. The authors concluded that both transradial and transbrachial approaches can be good alternative access routes when TFA is not appropriate in various neurointervential procedures [28]. 

Local complications associated with transbrachial approach were reported until now, more frequent in percutaneous coronary intervention. In a study conducted by Kiemeneij et al. [29], the risk of complications (median nerve palsy and pseudoaneurysm due to subcutaneous hematoma in the puncture site) associated with brachial artery access was 2.3%, a higher risk than that of the transfemoral approach. Webber et al. [30] observed that inserting a larger than 8-Fr sheath might increase the frequency of pseudoaneurysms. With our patient, no local complication due to TBA was observed. 

Small studies on transbrachial mechanical thrombectomy in acute ischemic stroke [3,31,32] concluded also that there were no technical difficulties or complications with this technique. Only a study conducted by Lu et al. on the safety and efficacy of the transbrachial approach for endovascular thrombectomy that included only 19 patients who had undergone MT for acute large vessel occlusion stroke concluded that the total procedure duration tended to be longer when the TBA was used after failure of the TFA (n = 6, 32%, median: 60.5 min) than when the TBA was used as the first side approach (n = 13, 68%, median: 22 min). Moreover, local complications were observed only in 2 cases: in one, a brachial artery pseudoaneurysm, and in the other, a brachial artery occlusion [3].

Regarding other access sites, mechanical thrombectomy via transradial and/or transbrachial approach in patients with AIS has recently been reported for both anterior and posterior circulation in the literature [27,33,34]. A systematic review and meta-analysis conducted by Kobeissi et al. that examine the feasibility of and outcomes following a transradial artery approach for posterior circulation large vessel occlusion strokes, observed a successful recanalization in almost all cases (98.69% rate, 93.50 to 100 cases) and a pooled meantime of puncture to recanalization of 29.19 min (24.05 to 35.42), concluding that transradial artery access for mechanical thrombectomy for this type of stroke displays early promise and feasibility [33]. After retrospectively analyzing five cases where the transbrachial approach was used for performing MT in acute stroke, Tsuji et al. observed that successful reperfusion was achieved in four out of five cases, and no access-site complications associated with this approach were reported in any case; however, death due to symptomatic intracranial hemorrhage was recorded [27]. When the transradial approach was compared with the transfemoral one, analyzing a homogeneous population in terms of the approach, which included a total of 2161 patients undergoing mechanical thrombectomy (446 patients that performed MT by transradial approach and 1715 patients by transfemoral approach), no significant differences across the two groups were found in terms of successful recanalization (*p* = 0.36), complete recanalization (*p* = 0.73), access-to-reperfusion time (mean difference, −3.92 min; *p* = 0.17), or symptomatic intracranial hemorrhage (*p* = 0.62). However, regarding access site complications, a significantly lower frequency in the transradial approach group was observed compared with the TFA group (*p* = 0.001) [35]. A low complication rate associated with transradial access, with only 1.4% ± 0.7% of stroke cases, was also noted by Peterson et al. after analyzing a number of 309 patients in which MT was performed via transradial approach from the studies included in their meta-analysis. Furthermore, when comparing studies that used the transradial approach with contemporary randomized clinical trials that used standard transfemoral access, no significant differences were found in puncture-to-reperfusion time, mortality, and/or site complications access of the transradial approach [36]. 

Two other alternative methods are available for patients who have experienced failed access for intracranial interventions: transcervical [37] (direct carotid puncture) and the recently introduced transvenous/transseptal access [38] (used to access the supra-aortic arteries from the venous side in cases where traditional transarterial access pathways, such as transfemoral, transradial/brachial routes, or direct carotid puncture, are expected to be unsuccessful). However, it is essential to note that direct carotid puncture carries the risk of serious complications such as neck hematoma, which could potentially compromise the airway. On the other hand, the two last methods, in emergency situations like AIS reperfusion, can be challenging. Transvenous/transseptal access presents logistical challenges and would not be suitable for patients with aortic pathologies, especially in aortic coarctation, as it would still require catheter manipulation in the aorta. The direct carotid approach in patients with AIS and coarctation of the aorta has been reported as a successful procedure in the literature so far, only by Roche et al. [39], who performed direct right common carotid puncture following the administration of the intravenous tissue plasminogen activator, in a 73-year-old woman with a large aortic arch saccular aneurysm measuring approximately 8 cm. But, also this approach technique, has disadvantages for patients with acute ischemic stroke. The primary issue commonly encountered with the cervical direct access technique is post-removal bleeding from the puncture site. To prevent the formation of cervical hematomas and the potential for obstruction of the upper airway, it is advisable to administer protamine after the procedure, apply manual compression, and, in some instances, utilize hemostatic closure devices. 

After performing MT via TBA in our patient case and reviewing the literature (see Table 1) [16,17,18,19,20,21,22], both TBA advantages and disadvantages for patients with acute ischemic stroke can be outlined. One drawback of this approach, in comparison to the transradial approach, is that the brachial artery is functionally an “end artery”, meaning that its blockage can lead to ischemia in the upper extremity. Fortunately, a surgical treatment is available for this condition, which can be performed immediately after intracranial thrombectomy. Additionally, based on our experience, achieving hemostasis is more challenging with the brachial approach than the radial approach. However, it is essential to note that the brachial artery is larger than the radial artery and has a lower risk of occlusion, mainly when the procedure is conducted under anticoagulation. Another advantage of the brachial approach is that it allows the operator to use shorter sheaths due to the closer proximity to the origins of the supra-aortic significant trunks [3]. 

Mechanical thrombectomy through the transbrachial approach was the first choice in more than half of these literature cases [18,19,20,22] (55.55%, n = 5 cases) in the treatment of acute ischemic stroke due to the presence of previously diagnosed aortic pathologies. In one-third of these cases (33.33%, our case and 2 case reports in the literature) [16,17], the transbrachial approach was chosen after attempting to advance the guiding catheter through the transfemoral approach and intraprocedural diagnosis of aortic pathology. In only one case [21], a patient with acute aortic dissection, after an ultrasound evaluation of the radial artery that showed a monophasic flow that could have caused difficulties and complications during the procedure, MT was performed via TBA. Local transbrachial complication was reported in one case [18], and in two other cases, it was not stated if there were such complications [19,21]. Hemorrhagic transformation of AIS was reported in two cases that underwent MT-only cerebral reperfusion via TBA, one with acute aortic dissection type A [19] and our case of previously undiagnosed aortic coarctation. In the cases in whom short and long-term follow-up was reported, the outcome of treatment, which was not exclusively endovascular (77.77% cases with only MT and 33.33% with association of first thrombolysis and after MT), was good (six from nine patients). In two case reports, the outcomes were not stated, and one patient died after a long hospitalization in the intensive care unit from respiratory complications (our patient).

In our case, the transbrachial approach allowed us to successfully rapidly reperfuse the patient’s brain, thus reducing access-to-reperfusion time. So, we advise that the emergency imaging of patients with acute stroke should include the assessment of the aortic arch and that it be analyzed in detail, especially in very young patients or elderly who may have aortic pathology. Our case and also the other 2 cases [16,17] in the literature in which the switch to alternative access was imposed by previously undiagnostic aortic pathology have emphasized the fact that neurointerventionalists must be familiar with alternative access routes because an urgent need for such access may occur during acute AIS treatment.

### Limitations

However, some issues may affect the generalizability of our findings. Compared to other large studies that included all patients with AIS and MT via transbrachial approach [3], our analysis only included case reports of patients with AIS and MT via TBA and aortic pathologies. Because there are several alternative approaches for patients with AIS, we believe that a larger study that includes a comparison between all these approaches already used in clinical practice in patients with AIS would help to choose the better approach for these types of patients, as well as studying the causes and risk factors that would affect the outcomes. For example, we cannot exclude the possibility that another approach type for MT is superior to TBA in patients with aortic pathology and AIS. 

## 4. Conclusions

The MT through the transbrachial approach is a treatment option that should be considered as the first choice, especially in aortic coarctation patients, where the transfemoral approach is difficult due to anatomical issues. Interventional neuroradiologists should familiarize themselves with the transbrachial approach, especially as the use of MT is increasing during the acute phase treatment of stroke. Moreover, during MT, it is crucial to optimize all strategies that have the potential to prevent delays and ensure prompt initiation of stroke treatment. 

In addition, in young hypertensive patients, unresponsive to antihypertensive treatment and admitted to the emergency department with acute stroke symptoms, we believe that the identification of an aortic pathology is essential. However, whether these patients should be routinely screened for aortic pathologies remains a matter of debate.

## Figures and Tables

**Table 1:** Summary of case reports of patients with acute ischemic stroke and aortic pathologies who underwent mechanical thrombectomy via transbrachial approach.

Number of Case Reports	Authors/Year	Sex/Age	Artery Occlusion Location	Reperfusion Therapy	Aortic Pathologies	MT Complications	Transbrachial Approach Local Complications	Outcomes
1.	Okawa et al., 2016 [16]	Not stated	Left terminal internal carotid artery	MT	Bovine aortic arch variant	No	No	Not stated
2.	Yamaguchi et al., 2017 [17]	Male, 94 years	Right middle cerebral artery	MT	Aortic arch type III	Hemorrhagic infarction of basal ganglia	Not stated	Alive, partially recovered
3.	Balci et al., 2019 [18]	Male, 57 years	Left middle cerebral artery m1 segment	MT	Thrombus protruding into the aortic arch	No	No	Alive, partially recovered
		Male, 60 years	Right middle cerebral artery m1 segment	MT	Thrombus protruding into the aortic arch	No	Moderate focal spasm at the entry site	Alive, partially recovered
4.	Kehara et al., 2020 [19]	Male/55 years	Right middle cerebral artery	MT	Acute aortic dissection type A	Right-sided hemorrhagic stroke	Not stated	Alive, recovered
5.	Proença et al., 2020 [20]	Male, 47 years	Mid-basilar artery	Intravenous thrombolysis + MT	History of type A aortic dissection (with previous endovascular stent graft surgery), intervention in the ascending and hemiarch aortic segment, ascending aortic aneurysm	No	No	Alive, recovered
6.	Bhatia et al., 2022 [21]	Female/51 years	Left middle cerebral artery	MT	Aortic bifurcation occlusion	Not stated	Not stated	Not stated
7.	Shimizu et al., 2023 [22]	Female/58 years	The left middle cerebral artery	Intravenous thrombolysis + MT	Aortic arch type III and Marfan syndrome	occlusion of the right internal carotid artery	No	Alive, partially recovered

## Data Availability

The data presented in this study are available on request from the corresponding author.
